# Hemogram parameters in the patients with subacute thyroiditis

**DOI:** 10.12669/pjms.36.2.1063

**Published:** 2020

**Authors:** Hasret Cengiz, Ceyhun Varim, Taner Demirci, Sedat Cetin

**Affiliations:** 1 Hasret Cengiz, Medical Doctor, Department of Endocrinology, Sakarya University Medicine Faculty, Sakarya, Turkey; 2 Ceyhun Varim, Associate Professor, Department of Internal Medicine, Sakarya University Medicine Faculty, Sakarya, Turkey; 3 Taner Demirci, Medical Doctor, Department of Endocrinology, Sakarya University Medicine Faculty, Sakarya, Turkey; 4 Sedat Cetin, Medical Doctor, Department of Endocrinology, Sakarya University Medicine Faculty, Sakarya, Turkey

**Keywords:** Subacute Thyroiditis, Hemogram, Neutrophil to Lymphocyte Ratio, Platelet to Lymphocyte Ratio, Mean Platelet Volume

## Abstract

**Background &Objective:**

Subacute Granulomatous Thyroiditis (De Quervain’s Thyroiditis) is an acute painful inflammatory disease of the thyroid. We aimed to investigate easily accessible and cheap hemogram based parameters of Neutrophil to Lymphocyte Ratio (NLR), Platelet to Lymphocyte Ratio (PLR), and Mean Platelet Volume (MPV) in the follow up of inflammatory thyroid disease.

**Methods:**

Patients admitted to Sakarya University Education and Research Hospital Endocrinology and Metabolism Outpatient Clinic and diagnosed as Subacute Granulomatous Thyroiditis between May 2017 and November 2018 were included in the study. Hemogram, thyroid function tests and acute phase values of these patients were recorded and compared with the values after treatment and disease recovery. On the sixth month, thyroid function tests were repeated and the rate of permanent hypothyroidism was screened. The relationships between initial hemogram parameters and acute phase reactants were evaluated.

**Results:**

Total 71 patients were included in our study. 60 (84.5%) were female and 11 (15.5%) were male. The F/M ratio was found to be 6/1. Mean age was 43 ± 9.95 years. Receiver Operating Characteristics (ROC) Curve Analysis was performed and values for Area Under the Curve (AUC) for NLR and PLR, respectively, were 0.739 (95% CI 0.657-0.820 p<0.0001) and 0.772 (95% CI 0.694-0.850 p<0.0001), which are significant and associated with disease activity. However, the AUC for MPV parameter was: 0.578 (95% CI 0.484-0.672 P_:_ 0.10) and was not significant. The cut off values defined as 2.4 (80% sensitivity and 51% specificity) for NLR and 146.84 (83% sensitivity and 54% specificity) for PLR for the acute phase of the disease. In the Correlation Analysis, NLR and PLR values were significantly correlated with ESR and CRP parameters, which are the most commonly used acute phase reactants.

**Conclusion:**

According to the present study, we believe that the NLR and PLR parameters will be of benefit in the follow-up the disease, accurately demonstrate the inflammatory load in the acute phase of the disease, and correlate with the common acute phase reactants.

## INTRODUCTION

Subacute granulomatous thyroiditis (De Quervain’s Thyroiditis); is an inflammatory thyroid disease with unknown etiology. The disease was first described by Mygind in 18 cases of ‘thyroiditis acuta simplex’ in 1895. In 1904, the Swiss Surgeon Fritz De Quervain clearly identified and separated the disease pathologically from the other inflammatory diseases of the thyroid^1^ developing usually within 2-3 weeks following a viral upper respiratory tract infection, and increasing in the frequency of seasonal passages, suggests that it is of autoimmune origin. Viral factors have also been blamed for its etiology, but the definitive etiologic factor has yet to be determined.

The diagnosis of the disease is made by combining clinical and laboratory data. It usually includes a severe inflammatory episode accompanied by significantly elevated Erythrocyte Sedimentation Rate (ESR) and C Reactive Protein (CRP) levels. Symptoms usually include severe neck pain, weakness, muscle and joint pains, and fever that rises above 38.5°C. Erythrocyte Sedimentation Rate (ESR) usually rises above 50 mm/h and can sometimes be seen above 100 mm / h. It is one of a few rare diseases that elevates ESR above 100mm/h in Internal Medicine Clinical Practice.

Neutrophil Lymphocyte Ratio (NLR) and Platelet Lymphocyte Ratio (PLR) are currently popular parameters used in the study of many diseases ranging from acute and chronic inflammatory and infective, respiratory and cardiovascular diseases to solid malignancies and hematological malignancies.

The effects of NLR and PLR and Mean Platelet Volumes (MPV) on the prognosis, survival and morbidity parameters on many inflammatory diseases and malignancies have been investigated. Significant correlations were found in many studies.^2^-^8^

Although its origin is suspicious, inflammatory activity is very obvious in this disease. The aim of this study was to investigate the value of these easily accessible and easily repeatable parameters in this inflammatory disease.

## METHODS

Seventy-one patients admitted to the Sakarya University Education and Research Hospital Endocrinology and Metabolism Outpatient Clinic and diagnosed as Subacute Granulomatous Thyroiditis between May 2017 and November 2018 were included the study. Demographic data of patients, fT4 (Thyroxin), TSH (Thyroid Stimulant Hormone), CRP, ESR, ALT (Alanine Aminotransferase), AST (Aspartate Aminotransferase), Creatinine, WBC (White Blood Cell), Lymphocytes, Neutrophil, Mean Platelet Volume (MPV) and Platelet Values were recorded. After complete resolution of the disease by treatment, in the same laboratory parameters and acute phase reactants were again recorded to check correlation with disease activity. The dose and duration of NSAIDs (nonsteroidal anti-inflammatory drugs), thionamides, steroids, antibiotics and beta-blockers were also recorded. Thyroid function tests (fT4, TSH) were then used to detect permanent hypothyroidism at the six-month follow-up.

Female and male subacute thyroiditis patients over 18 years of age were included in the study. Patients with acute or chronic infections, inflammatory and rheumatic diseases that may affect blood parameters and acute phase reactants, and those who have used steroids in the last who months, pregnant women, and those with chronic renal failure, chronic liver disease, congestive heart failure, hematological malignancy or solid organ malignancy were not included in the study. Local ethics committee approval (Ref. No.: 71522473/050.01.04/334) was obtained from the Sakarya University Ethics Committee.

### Laboratory parameters

Blood samples were taken after eight hours fasting in the morning during diagnosis and follow-up, sent to the laboratory immediately and centrifuged at 2000 rpm for 15 minutes. For biochemical parameters the samples were placed into a dry tube and investigated using a Beckman Coulter AU680® with Beckman Coulter kits. Blood was placed into the EDTA tube for a hemogram examination via a WIC-LYSE for CELL DYN 3700 Kits on the Abbott Cell-Dyn 3700® Device. ESR was performed with Rapida ESR100® in capillary tubes. The CRP parameter was studied with SIEMENS BN II^®^ with Cardio Phase hsCRP WN^®^ kits.

TSH and fT4 parameters were studied with Abbott Architect I 2000 SR®. The data was automatically uploaded to the hospital database system and screened from there. MPV (N: 7.5-11.5 femtoliter) values were recorded from the measured value in the hemogram.

Neutrophil Lymphocyte ratio was calculated by dividing absolute neutrophil count by absolute lymphocyte count, and Platelet lymphocyte ratio was calculated by dividing absolute platelet count by absolute lymphocyte count.

### Statistical Analysis

Data analysis was performed by using SPSS for Windows 24 (Statistical Package for Social Science, SPSS Inc. Chicago IL, USA ^®Z^). Mean differences of continuous data between paired samples were evaluated by Paired Sample t-Test, while the Wilcoxon Signed-Rank Test was used to analyze noncontinuous variables. Chi-Square Test was used to compare differences between categoric variables, and Receiver Operating Characteristic Curves (ROC) were conducted to find out cut off values for NLR, PLR and MPV parameters. The statistically significant two tailed p-value was considered as <0.05.

## RESULTS

Among the seventy-one patients included in our study, 60 (84.5%) were female and 11 (15.5%) were male. F/M ratio found as 6/1. The mean age was 43 ± 9.95 years (max: 81 years, min: 26 years).

During the acute inflammatory phase of the disease, mean ESR was 74 ± 27.7 mm / h (N: <50 years <20 mm / s> 50 years <30 mm / s), CRP: 44 ± 38.8 mg / L (N: <5 mg / L), TSH: 0.34 ± 0.63 µU / L (N: 0.34-4.6)µU /L), fT4: 17.6 ± 4.37 pmol / L (N: 8.9-19.9 pmol / L), MPV: 7,5 ± 1,25 fL (N: 7,5-11,5 fL), NLR: 2,78 ± 1,52 and PLR: 173,04 ± 74. At the resolution phase of disease the values were: mean ESR: 25.5 ± 15.5 mm / h CRP: 6.39 ± 7.3 mg / L TSH: 3.3 ± 2.6 µUL fT4: 12.2 ± 3.5 pmol / L MPV: 7, 9 ± 1.19 fL NLR: 1.9 ± 1.06 and PLR was 118.3 ± 37.5 (Table-I).

**Table-I:** Laboratory parameters in acute period and recovery period.

	Acute nflammatory Phase of the Disease	Recovery Phase of the Disease	Sig. (P value)	r Value
ESR (mm/h)	74±27.7	25.5±15.5	<0.0001	0.222
CRP (mg/L)	44±38.8	6.3±7.3	<0.0001	0.132
TSH (µU/L)	0.34±0.63	3.3±2.6	<0.0001	-0.155
fT4(pmol/L)	17.6±4.37	12.2±3.5	<0.0001	0.094
Wbc (10^3^/mm^3^)	8.37±2.07	7.36±1.78	<0.0001	0.054
Neu (10^3^/mm^3^)	5.3±1.8	4.2±1.5	<0.0001	0.086
Lym (10^3^/mm^3^)	2.1±0.63	2.4±0.69	<0.0001	-0.074
Plt (10^3^/mm^3^)	343±103.4	272.04±64.9	<0.0001	0.208
MPV (fL)	7.5±1.25	7.9±1.19	0.12	-0.119
NLR	2.78±1.52	1.9±1.06	<0.0001	0.422
PLR	173.04±74	118.3±37.5	<0.0001	0.598

For treatment, NSAID was used for a median 2.8±2.1 weeks, Steroid Therapy was used for a median 1.8±2.69 weeks, antithyroid treatment was used for a median 0.57±1.64 weeks, antibiotic treatment was used for a median 0.22±0.59 weeks, beta blocker treatment was used for a median 1.11±1.60 weeks.

**Fig.1:**
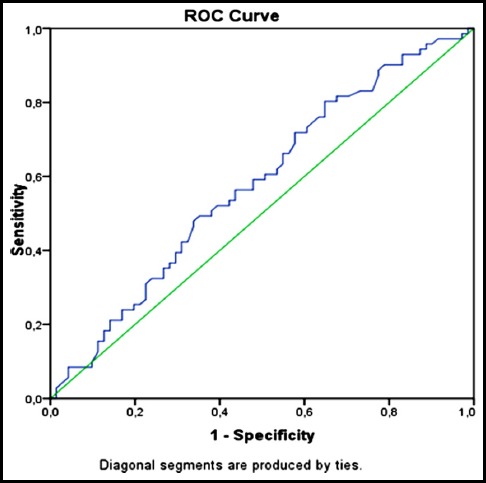
ROC Curve Analysis for MPV (AUC): 0,578 (%95 CI 0,484-0,672 P_:_ 0,10).

**Fig.2:**
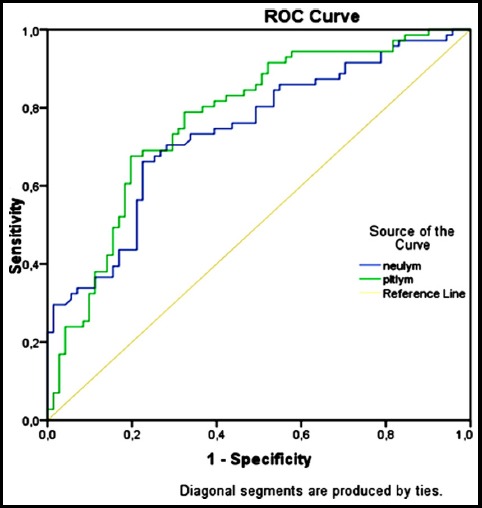
ROC Curve Analysis for NLR and PLR Parameters.

Receiver Operating Characteristics (ROC) Curve Analysis was performed for NLR, PLR and MPV parameters. For MPV Area, the Under the Curve (AUC) value was: 0.578 (95% CI 0.484-0.672 P_:_ 0.10) and was not significant (Fig.1).For NLR and PLR parameters AUC values were respectively: 0.739 (95% CI 0.657-0.820 p<0.0001) and 0.772 (95% CI 0.694-0.850 p<0.0001) were found to be significant and associated with disease activity (Fig.2, Table-II).

**Table-II:** AUC Values for the NLR. PLR and MPV parameters.

	AUC	%95 CI	P Value
NLR	0,739	0,657-0,820	<0,0001
PLR	0,772	0,684-0,850	<0,0001
MPV	0,578	0,484-0,672	0,1

The descriptive cut off value of NLR was 2.4 (80% sensitivity and 51% specificity), and of PLR was 146.84 (83% sensitivity and 54% specificity) for the acute inflammatory phase of the disease (Fig.2). In the correlation analysis, NLR and PLR values were significantly correlated with ESR and CRP parameters in inflammatory phase, which are the most commonly used acute phase reactants. (P: 0.008 r:0,313 for NLR-ESR, p: 0.001 r:0,398 for NLR-CRP, p: 0.001 r:0,374 for PLR-ESR, P <0.001 r:0,459 for PLR-CRP). However, these correlations disappeared during the resolution phase of the disease. (P: 0.78 r:0,026 for NLR-ESR, p: 0.74 r:0,204 for NLR-CRP, p: 0.59 r:0,171 for PLR-ESR and p: 0.57 r:0,244 for PLR-CRP).In addition, there was a negative and significant correlation between ESR and TSH in the active period of the disease (p: 0.006 r:-0,323) but no significant correlation was found during the recovery period.

When we examined these parameters for association with long-term hypothyroidism, no significant correlation was found between long-term hypothyroidism and initial NLR, PLR and CRP values (p values: 0.74-0.73-0.38, respectively). However, significant association between initial ESR and permanent subclinical hypothyroidism was found (p: 0.03) (Table-I).

## DISCUSSION

Subacute granulomatous thyroiditis is a rare painful inflammation of the thyroid. Its prevalence varies between 3-12 /100 000 by various sources. It accounts for approximately 5% of clinically evident thyroid diseases. The disease is more common in women, with a F/M ratio is around 4-7 / 1. It is usually seen in the 40s and 50s.^9^-^12^ It mostly develops in summer and autumn, usually after 2-3 weeks following a viral upper respiratory infection, with the clinic of neck pain, weakness, chills, fever, diffuse muscles and joint pain. Although viral factors such as Coxsackievirus, Adenovirus, and Influenza virus infection are considered in the etiology, no definitive factor has been determined yet.^13^,^14^ In addition, 70% of the cases are HLA B35 positive.^15^ Diagnosis is made by clinical, laboratory and imaging findings. On the Acute period of disease; extremely high acute phase reactants such as ESR and CRP is usually seen together with a subclinical hyperthyroidism. Thyrotoxicosis develops for 2-4 weeks with the release of large amounts of thyroid hormone as a result of the inflammatory damage to the thyroid follicles. Anti-thyroid peroxidase and anti-thyroglobulin antibodies are generally normal.^16^ Ultrasonography shows diffuse enlargement of the thyroid dimensions, and hypoechoic patchy nodulation areas. There is no increase in thyroid blood supply in Doppler Ultrasonography. Iodine and technetium uptake in the scintigraphy is below 5%.^17^ Thyroid biopsy is not used in routine for diagnosis. When biopsies are performed on uncertain cases, follicular and thyroid cell destruction in the acute phase, diffuse polymorphonuclear leukocytes infiltration in the subacute phase diffuse lymphocyte, and mononuclear cell infiltration and giant cell granulomatous inflammation are found.^9^ Even if the disease is not treated, it tends to resorb in 2-8 weeks spontaneously. Thyroid wrecking because of severe inflammation results in a transient hypothyroidism which lasts generally 2 to 8 weeks. However, patients usually become euthyroid at around 6 months. In some rare cases, permanent hypothyroidism development can occur after 12 months. Permanent hypothyroidism develops in 5% of the patients and is usually seen around 6 months. About 2% of patients develop recurrence.^18^,^19^

As shown, Subacute Granulomatous Thyroiditis has a very high inflammatory load with very high ESR and CRP levels, severe clinic, and histological verifications by previous studies. Therefore, we needed to study the parameters of NLR and PLR, which are the most easily accessible parameters that have been studied in many inflammatory and non-inflammatory diseases.

High neutrophil lymphocyte ratio is due to both increased absolute neutrophil count and decreased absolute lymphocyte count. Neutrophil activation abnormalities have been previously studied in many autoimmune diseases and in the pathogenesis of tissue destruction in several studies. Neutrophils are primarily elements of innate immune system, whereas lymphocytes are elements of an adaptive immune system. Adaptive immune system defects have been studied in the pathogenesis of many autoimmune conditions too. Immunoregulation disorder with early apoptosis of lymphocytes may be a mechanism that triggers autoimmunity.

Platelets are normally hemostasis cells. However, as evidenced in recent studies, platelets have regulator effects on both innate and adaptive immune systems. It is now evident that various cytokines from platelets and neutrophils activate the innate and adaptive immune system, which is caused by vicious cycle more and more neutrophil and platelet activation, causing tissue destruction. This is involved in the pathogenesis of many acute and chronic inflammatory and autoimmune conditions.^20^-^22^

In our study, we investigated whether NLR, PLR and MPV parameters can be used in the diagnosis and follow-up of patients with Subacute Granulomatous Thyroiditis. The NLR and PLR values were positively correlated with the most common acute phase reactants such as ESR and CRP on the acute phase, and were found to be significantly higher in the active period of the disease. For the acute phase, we determined an NLR cut off value of 2.4 and PLR cut off value of 146.8 with acceptable sensitivity and specificity. No significant correlation or cut off value was found for MPV parameter. The correlation between NLR and PLR with ESR and CRP was disappeared after the disease was completely recovered. This showed us that these two parameters can be used as an additive to the acute phase reactants in detecting the inflammatory load at the time of diagnosis. In the present study, NLR and PLR’s relationships with permanent subclinical hypothyroidism were also examined, but no significance was detected. Only the acute period ESR and persistent subclinical hypothyroidism were found to be significantly related.

Neutrophil Lymphocyte Ratio and PLR have been studied as diagnostic and prognostic factors in many diseases, including acute and chronic inflammatory and infectious diseases, respiratory system diseases, cardiovascular system diseases and even many cancer types (2-8). For the thyroid, Chronic Autoimmune Lymphocytic Thyroiditis, (Hashimoto Thyroiditis) and thyroid cancer related studies are available. In Hashimoto Thyroiditis patients; NLR and PLR values were found to be significantly higher than euthyroid controls and correlated with acute phase parameters like our study.^23^,^24^ In a third study, NLR and PLR were found to be significantly higher in Hashimoto Thyroiditis patients compared to healthy controls and correlated with Thyroid Autoantibodies. The MPV parameter was also examined but no significant differences were found as in our study.^25^

Neutrophil to Lymphocyte Ratio and PLR in Thyroid Cancer have been investigated in many studies as well.^26^-^29^ Significant correlations between NLR and PLR and prognostic factors were recorded in most of these studies.

When the literature was screened, two previous studies on Subacute Granulomatous Thyroiditis were found. The first one was published in China in 2017. NLR and PLR parameters were examined in patients with thyrotoxicosis. Among 169 subacute granulomatous thyroiditis patients a subgroup had significantly higher NLR and PLR values (p <0.01), and positive correlations were found for NLR with ESR and WBC and PLR with ESR and fT4. According to the ROC Curve Analysis, the cut-off value of the acute phase of Subacute Thyroiditis for NLR was found to be 2.0 (sensitivity 80.5%, specificity 76.9% AUC 0.833) and PLR was found to be 150 (sensitivity 64.3%, specificity 84.2% AUC 0.801).^30^

In the second study, also conducted in Turkey, seventy-five subacute granulomatous thyroiditis patients were compared with 75 healthy controls and only examined for the NLR parameter. As a result of this study, NLR (3.56 ± 2.64) was significantly higher in Subacute Granulomatous Thyroiditis group than the control group (1.41 ± 0.9). They also observed significant elevations in ESR and CRP values in the patient group compared to the control group, but the correlations of these parameters with NLR and PLR were not studied.^31^ We have not yet come across any study in the literature on subacute thyroiditis and MPV, which is an indicator of platelet activation. However above mentioned study from Arpaci D et al. studied MPV parameter in Hashimoto Thyroiditis but had found no significance.^25^

### Limitations of the study

This is a single-center study is a relatively small number of cases and a limited follow-up period. For example, sometimes the development of permanent hypothyroidism may extend to 12 months, but unfortunately, we were unable to get 12 months of data for most of our patients.

Subacute thyroiditis etiology has not been determined yet, but fortunately it is a self-limiting disease of benign nature. However, in the acute period, the strong inflammatory stress of the disease is exhausting for the patient and physician. Studies on finding additional parameters to guide the management and treatment of this inflammatory process are ongoing. However, more comprehensive studies are needed to better understand the etiopathogenesis of the disease and follow-up parameters.

## CONCLUSION

We found that NLR and PLR values were significantly higher during the acute period of the disease in patients with Subacute Granulomatous Thyroiditis. High correlation with the common acute phase reactants and normalization with disease resolution showed that these parameters could provide additional benefit in the follow-up and correct treatment approach in the acute phase of the disease. These cheap, easily accessible and repeatable parameters in diagnosis and follow-up will facilitate the work of clinicians.

### Authors’ Contribution:

**HC:** Conceived the study and found, enrolled patients, is responsible for integrity of research.

**CV:** He made statistical analysis and made the last controls.

**TD:** He wrote the article.

**SC:** He translated the article to the English and prepared the tables.
